# Improving Clinical Validity in Synthetic Electronic Health Record Generation Using Best-of-N Sampling: Comparative Evaluation Study

**DOI:** 10.2196/90590

**Published:** 2026-07-30

**Authors:** Md Akmol Masud, Mahmud Hasan

**Affiliations:** 1 Department of Electrical and Computer Engineering, Queen's University, 99 University Ave, Kingston, ON, K7L 3N6, Canada, +880 1304963440; 2 Department of Biostatistics, Virginia Commonwealth University, Richmond, VA, United States

**Keywords:** synthetic data generation, electronic health records, generative adversarial networks, clinical validity, inference-time selection, constraint satisfaction, tabular data synthesis, valid support mass

## Abstract

**Background:**

Synthetic electronic health record generation is limited not only by statistical fidelity but also by clinical validity. Records that appear statistically plausible may still violate hard structural, physiological, or relational constraints.

**Objective:**

This study evaluated best-of-N constraint-minimizing selection as an inference-time strategy for improving clinical validity and characterized when such selection succeeds or fails as a function of the generator’s valid support mass (*p*_valid_).

**Methods:**

We evaluated Wasserstein generative adversarial network with gradient penalty (WGAN-GP) and conditional tabular generative adversarial network (CTGAN) on 3 public clinical tabular datasets: stroke prediction (n=5110), diabetes health indicators (n=100,000), and cardiovascular disease (n=68,599). For each generator, we compared random sampling, naive clipping, and best-of-N selection (N ∈ {8, 16, 128}). Validity was assessed via dataset-specific rule violations; fidelity via Kolmogorov-Smirnov (KS) statistics and correlation preservation; utility via TSTR (train-on-synthetic-test-on-real) AUC (area under the receiver operating characteristic curve); and privacy via membership inference attack (MIA) AUC and distance to closest record (DCR).

**Results:**

Best-of-N effectiveness was governed by the base generator’s valid support mass. In stroke, WGAN-GP had *p*_valid_=0, and all selection strategies, including strict rejection across 5000 draws, produced 500/500 (100%) invalid samples. In cardiovascular data, WGAN-GP had *p*_valid_=0.12; best-of-16 reduced violations from 441/500 (88.2%) to 65/500 (13.0%), and best-of-128 eliminated them entirely (0/500), matching theoretical predictions exactly. In diabetes, WGAN-GP random sampling was already fully valid (0/500 violations), and best-of-N primarily shifted utility. Across all 3 datasets, CTGAN+best-of-16 achieved 0/500 violations with strong fidelity (mean KS≤0.12) and utility (TSTR AUC up to 0.81). MIA AUC remained near-random guessing (approximately 0.50) throughout, though DCR-based proximity concentration increased markedly under CTGAN selection (reaching 22.6% on stroke and 100% on cardiovascular data).

**Conclusions:**

Best-of-N functions as a bounded-budget feasibility filter rather than a universal repair mechanism. Its effectiveness depends critically on the generator’s valid support mass. CTGAN+best-of-16 offered the strongest overall trade-off across clinical validity, fidelity, utility, and privacy in our experiments.

## Introduction

### Best-of-N Sampling

The digitization of health care has created vast repositories of electronic health records (EHRs) with significant potential to accelerate medical research and improve patient outcomes. However, this potential remains largely untapped due to stringent privacy regulations (eg, HIPAA [Health Insurance Portability and Accountability Act] and the GDPR [General Data Protection Regulation]) that restrict data sharing. Generative adversarial networks (GANs) [1] and newer tabular generators are widely used to create synthetic “shadow datasets” that can preserve important statistical characteristics while reducing direct data-sharing burden, although privacy risk must still be evaluated explicitly [2-5]. A more rigorous theoretical foundation for GAN-based modeling, including error and divergence analyses, can be found in the study of Hasan and Sang [6,7].

However, transitioning from research prototypes to real-world applications can be quite challenging, primarily due to concerns about clinical validity. Unlike natural images, where minor artifacts may be acceptable, clinical tabular records are governed by strict structural, physiological, and relational rules. A synthetic record may align with marginal distributions yet be impossible or clinically incoherent. Modern tabular generators such as Wasserstein GAN with gradient penalty (WGAN-GP) [8] and conditional tabular GAN (CTGAN) [9] therefore pose a practical question that statistical fidelity alone cannot answer: when can invalid outputs be repaired by inference-time selection, and when is the problem already baked into the learned support of the generator?

This discrepancy arises because standard generators optimize for distributional similarity rather than rule compliance. Bridging this “validity gap” has traditionally required one of 2 extremes: increasing architectural complexity by embedding constraints directly into the model or loss [10] or naive postprocessing, where invalid values are clipped or rounded after generation [11].

### Study Aim

We study best-of-N sampling as a constrained selection strategy. For each desired output row, the method draws N candidates from the generator and returns the candidate with the minimum violation score. This mechanism is simpler than retraining and less destructive than clipping; however, it is not classical rejection sampling and cannot create valid samples when the generator assigns zero probability mass to the feasible region. We evaluate best-of-N across 3 datasets and 2 generator families and explicitly compare empirical success with the joint-validity probability *p*_valid_=P(V(X)=0). This paper makes the following contributions:

- We propose best-of-N as a constraint-minimizing selection method and evaluate it on stroke, diabetes, and cardiovascular tabular datasets.
- We show empirically that best-of-N success is governed by valid support mass: it fails completely when *p*_valid_=0 (stroke WGAN-GP), follows theoretical predictions when *p*_valid_ is moderate (cardiovascular WGAN-GP), and becomes largely unnecessary when validity is already high (diabetes WGAN-GP).
- We benchmark the same selection strategy on CTGAN and show that CTGAN+Best-of-16 achieves 0.0% violations across all 3 datasets, giving the strongest overall practical trade-off in our experiments.
- We provide sensitivity, rejection-sampling, and privacy-concentration analyses to clarify how constraint weighting, candidate budget, and base generator interact.

All codes, constraint checkers, and the full evaluation pipeline are openly available on GitHub [12].

### Related Work

#### Prior Approaches to Tabular Data Synthesis and Constraint Enforcement

The challenge of generating realistic tabular data has attracted significant attention in recent years. CTGAN [9] introduced mode-specific normalization and conditional generation to handle mixed data types, becoming a de facto baseline for tabular synthesis. Tabular variational autoencoder [9], its variational autoencoder counterpart, offers improved stability at the cost of sample diversity. More recently, diffusion-based approaches such as tabular denoising diffusion probabilistic model [13] have demonstrated competitive performance, particularly for datasets with complex feature interactions. Large language model (LLM)–based methods such as GReaT (Generation of Realistic Tabular data) [14] reframe tabular generation as text completion. Broader surveys and benchmarking frameworks now emphasize that utility, fidelity, alignment, and privacy should be evaluated jointly rather than in isolation [15-18].

Enforcing constraints in generative models has been approached from multiple angles. Architectural approaches embed constraints directly into the model structure or loss function. DC3 [10] introduces differentiable constraint layers that project outputs onto feasible regions, and recent tabular-specific constrained generative modeling extends this line by enforcing explicit rule compliance in deep generators [19]. However, these methods often complicate training dynamics and require constraint-specific architectures. Post hoc approaches apply corrections after generation. Simple clipping and rounding [11] guarantee validity but can distort distributions. Classical rejection sampling discards all invalid samples entirely, preserving distributional properties at the cost of efficiency. Our constraint-minimizing selection (best-of-N) approach ranks and selects the best of N candidates rather than discarding them outright, achieving high validity with bounded computational overhead.

Generating synthetic clinical data possess unique challenges due to privacy regulations and the need for clinical validity. MedGAN (medical generative adversarial network) [3] pioneered the application of GANs to EHRs, demonstrating feasibility but struggling to satisfy constraints. CorGAN (correlational generative adversarial network) [20] improved correlation preservation through convolutional architectures. Recent work by Yale et al [21] highlighted the tension between privacy guarantees and data utility in synthetic EHR generation. Our contribution addresses a gap in this literature. While prior work has focused on improving statistical fidelity or privacy, we systematically characterize the efficiency-validity trade-off for clinical constraints, providing practical guidance for deployment. We summarize the conceptual positioning of our proposed best-of-N selection strategy relative to these representative prior methods in Table 1.

**Table 1.** Positioning of the proposed best-of-N selection strategy relative to representative prior work.

Method	Inference time	Bounded budget	Formal *p*_valid_	Multidataset	Privacy audit
DC3 [10]	—^a^	—	—	✓^b^	—
Stoian et al [19] (2023)	—	—	—	✓	—
Naive clipping [11]	✓	✓	—	~^c^	—
Rejection sampling	✓	—	✓	~	—
MedGAN^d^ [3]	—	—	—	—	✓
CorGAN^e^ [20]	—	—	—	—	✓
Best-of-N (ours)	✓	✓	✓	✓	✓

a Not addressed.

b Addressed.

c Partially addressed.

d MedGAN: medical generative adversarial network.

e CorGAN: correlational generative adversarial network.

#### Connections to LLM Alignment and Monte Carlo Methods

Conceptually, our formulation situates tabular synthesis near 2 established statistical traditions. First, best-of-N constraint-minimizing selection corresponds exactly to inference-time best-of-N sampling in LLM alignment [22,23]. Under paradigms such as Group Relative Policy Optimization [24], models sample N candidates, evaluate them using a reward function, and leverage relative scores to guide selection or learning. Our approach mirrors this conceptual skeleton, sampling a group, evaluating it against a scoring function, and returning the optimal candidate, but focuses solely on post hoc feasibility selection rather than on policy updates via backpropagation. Second, our mechanistic analysis of valid support mass (*p*_valid_) and the success formula 1 − (1 − *p*_valid_)^N^ is rooted in classical Monte Carlo feasibility estimation. Best-of-N serves as a principled Monte Carlo geometric approach to clinical tabular generation.

## Methods

### Overview

The goal of this study is to evaluate a data synthesis pipeline that produces clinically valid samples without compromising privacy or downstream usefulness. Our approach consists of three stages: (1) rigorous problem definition via dataset-specific constraints, (2) training a tabular generator backbone (WGAN-GP or CTGAN), and (3) inference-time post hoc sampling through random draws, naive repair, best-of-N selection, or strict rejection. We evaluate our approach on 3 publicly available clinical datasets, summarized in Table 2. Each dataset was selected to represent a different tier of constraint complexity, ranging from simple univariate bounds to complex multivariate correlations. All datasets were partitioned into training (70%), validation (15%), and testing (15%) sets.

**Table 2.** Summary of clinical datasets and constraint tiers.

Dataset	N	Tier	Key constraints
Stroke prediction [25]	5110	Simple	One-hot gender consistency; BMI 10‐60 kg/m^2^; age 0‐120 years; if age <18 years, then not married
Diabetes health indicators [26]	100,000	Intermediate	Sex in {0,1}; BMI 10‐98 kg/m^2^; Diabetes_binary=1 inconsistent with GenHlth<1.5
Cardiovascular disease [27]	68,599	Complex	Valid gender encoding; ap_hi 60‐250 mm Hg; ap_lo 40‐200 mm Hg; ap_hi_ > ap_lo_+10 mm Hg

### Generator Backbones

We used 2 tabular generator families. First, we trained a WGAN-GP [8] as a deliberately simple baseline to study inference-time selection under imperfect validity. Second, we trained CTGAN [9] as a stronger tabular benchmark to test whether the same selection rule remains useful when the base model better captures mixed-type tabular structure. WGAN-GP models were trained with spectral normalization and a small soft-constraint penalty (λ_constraint_=0.1); CTGAN was trained with its standard conditional sampling mechanism. Additionally, a purely unconstrained WGAN-GP baseline (λ=0, no soft-constraint penalty) was included to isolate the contribution of inference-time selection from that of the penalty term.

### Inference Strategy: Best-of-N Selection

Best-of-N operates as a bounded-budget selection filter. For each desired synthetic record, we draw N candidate samples from a trained generator and return the candidate with the smallest violation score. This is not strict rejection sampling: the algorithm always returns the lowest-scoring candidate, even if all N candidates are invalid. Textbox 1 formalizes this procedure. We evaluated N ∈ {8, 16, 128} to capture bounded-budget, diminishing-returns, and large-budget regimes. For comparison, we also evaluated (1) random sampling, which draws directly from the generator without any filtering; (2) naive postprocessing, which applies deterministic value clipping, rounding, or one-hot repair after generation; and (3) strict rejection sampling, which repeatedly draws until *V*(*x*)=0, used to distinguish ranking-selection from true acceptance filtering.

Textbox 1. **Algorithm of best-of-N constraint-minimizing selection.**

Input: Trained generator G, number of candidates N, constraint checker V(·)

Output: A selected synthetic sample x*

1. for each desired output sample do
2. Sample N latent vectors: {z1,...,zN} ~ N(0,I)
3. Generate candidates: {x1,...,xN} <- G(z1),...,G(zN)
4. Evaluate violation scores: si <- V(xi) for all i
5. Select best candidate: x* <- argmin_i si
6. return x*

### Violation Score Function

The constraint checker computes per-record violations in 4 categories: structural or gender encoding, physiological range, relational correlation, and logical consistency. The default score is an equal-weight sum of indicator functions:

$V(x)=V_{\text{gender}}(x)+V_{\text{physio}}(x)+V_{\text{corr}}(x)+V_{\text{logic}}(x)$(1)

For stroke, *V*_gender_ enforces one-hot gender consistency: |gender_male_+ gender_female_ − 1| > 0.1 or out-of-range category values. For cardiovascular data, *V*_corr_ enforces the relational rule ap_hi_ > ap_lo_ + 10 mm Hg, and *V*_gender_ enforces valid encoding of the binary/ordinal gender field. For diabetes, *V*_corr_ encodes the dataset-specific logical inconsistency between diabetes status and self-reported general health. Equal weights are used because the primary objective is feasibility rather than ranking clinical severity; severity-weighted and no-gender ablations were also evaluated on stroke and cardiovascular data to assess robustness.

### Joint-Validity Interpretation

Let *p*_valid_ = *P*(*V*(*X*) = 0) denote the joint probability that a random sample from the trained generator satisfies all constraints simultaneously. Under independent draws:

$P(\text{at least 1 valid sample})=1-(1-p_{\text{valid}}{)}^{N}$(2)

We estimated *p*_valid_ empirically from raw generator samples:

${\hat{p}}_{valid}=\frac{1}{M}\sum_{i=1}^{M}1\{V(x_{i})=0\},\;x_{i}∼P_{model}$(3)

This yields the central distinction of the paper: when *p*_valid_=0, neither best-of-N nor strict rejection can recover valid rows; when *p*_valid_ is moderate, empirical gains should follow the formula above.

### Experimental Setup

All experiments were conducted in Python with PyTorch, Pandas (NumFOCUS, Inc), NumPy (NumFOCUS, Inc), SciPy (NumFOCUS, Inc), and scikit-learn (INRIA; French Institute for Research in Computer Science and Automation). For each dataset, we partitioned the data into training (70%), validation (15%), and test (15%) subsets and generated 500 synthetic samples per method for evaluation. WGAN-GP models used early stopping and a soft-constraint penalty of λ_constraint_ = 0.1; CTGAN was trained with its default conditional tabular formulation. Statistical uncertainty due to sampling variance was estimated using bootstrap resampling (B=1000 iterations). 95% CIs for violation rates are reported as Wilson score intervals based on 500 generated samples. Because algorithmic constraint matching is deterministic and inherently bounded by the 0/1 rule, formal *P* values are omitted; instead, we report absolute counts and empirical percentages throughout.

We report wall-clock time per generated sample for all main methods. For the mechanism analysis, we additionally report the expected time per valid sample, which is more informative than a kept/generated ratio when *N* is fixed by design. If a single draw takes an expected time *t*_g_, then strict rejection has an expected cost *E*[*T*_reject_]=*t*_g_/*p*_valid_ per valid sample, while bounded-budget best-of-N has approximate cost *E*[*T*_BoN_(*N*)] ≈ *Nt*_g_ / [1 − (1 − *p*_valid_)^*N*^]. These expressions explain why rejection fails when *p*_valid_=0, and why large *N* can become inefficient after validity saturates, as illustrated by the costs reported above.

### Ethical Considerations

The datasets used in this study were obtained from publicly available repositories on University of California Irvine Machine Learning and Kaggle. All records are deidentified; therefore, this study did not involve human participants and was exempt from institutional review board review. The original data collection for each dataset was conducted under institutional ethical oversight, and the public release terms explicitly permit unrestricted secondary analysis; no additional informed consent was required for this study. Privacy preservation was empirically verified using membership inference attack (MIA) metrics (MIA AUC [area under the receiver operating characteristic curve] of approximately 0.50 across all methods).

### Evaluation Metrics

The framework targeted 4 dimensions: validity (any-violation rate, target 0%), fidelity (Kolmogorov-Smirnov [KS] statistic, lower is better; correlation preservation, target≥0.90), utility (TSTR [train-on-synthetic-test-on-real] AUC, target≈real-data AUC), and privacy (MIA AUC≈0.50; distance to closest record [DCR] minimum ≥0). The computation methodology and formal targets for each metric are described in the following subsections.

Clinical validity was computed per record and summarized as the proportion of generated rows with at least one violation. Statistical fidelity was assessed using per-feature KS statistics and correlation preservation (the Frobenius norm of the difference between the synthetic and real Pearson correlation matrices). Utility was measured using TSTR AUC from a random forest classifier trained on synthetic data and evaluated on a held-out real test set. Privacy was assessed using MIA AUC and DCR summaries, with additional DCR-threshold concentration analysis for stroke and cardiovascular data. We use MIA as a standard empirical proxy for privacy attacks [28-31], following recent medical synthetic-data evaluations that recommend multimetric privacy reporting rather than a single attack score [5,18].

## Results

### Overview

The updated experiments revealed 3 qualitatively different regimes for inference-time selection: a zero-valid support regime, a mostly valid regime, and a moderate-valid support regime. These regimes, rather than the nominal complexity of the constraint list alone, determined whether Best-of-N selection was useful.

### Clinical Validity Across Datasets

**Figure 1.**
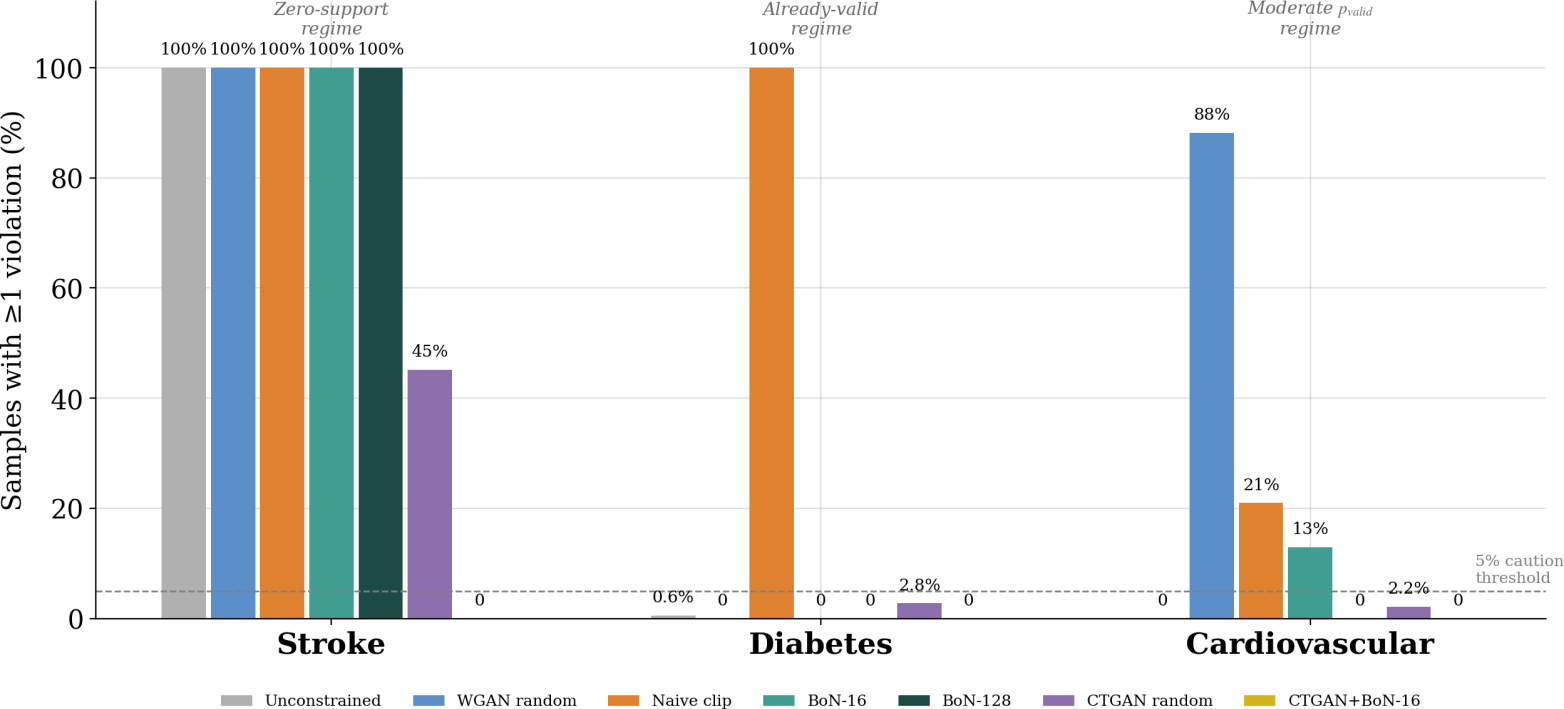
Cross-dataset violation rates by method, including the unfiltered conditional tabular generative adversarial network (CTGAN) random baseline. Each cluster of bars corresponds to one dataset, illustrating 3 regimes: a zero-support regime (stroke, where all Wasserstein GAN with gradient penalty [WGAN-GP] variants fail), an already-valid regime (diabetes, where WGAN-GP random sampling produces 0% violations), and a moderate-*p*_valid_ regime (cardiovascular, where best-of-N (BoN) tracks the theoretical improvement curve). Exact counts: stroke CTGAN random: 226/500 (45.2%); stroke CTGAN+BoN-16: 0/500; diabetes CTGAN random: 14/500 (2.8%); cardiovascular CTGAN random: 11/500 (2.2%); cardiovascular WGAN BoN-128: 0/500; diabetes naive clipping produces 100% violations due to binary feature distortion.

Figure 1 summarizes violation rates across all 3 datasets and the full method stack, revealing 3 qualitatively distinct regimes. On stroke, WGAN-GP had no valid support mass (*p*_valid_=0): random sampling, best-of-16, and best-of-128 all produced 500/500 (100%) invalid samples, and only CTGAN+best-of-16 reached 0/500 (0%) violations. On diabetes, WGAN-GP random sampling was already fully valid at 0/500 (0%) violations; the dataset illustrates the already-valid regime, where best-of-N shifts utility but not validity. On cardiovascular data, WGAN-GP random sampling produced 441/500 (88.2%, 95% Wilson CI 85.1%‐90.7%) invalid rows, best-of-16 reduced this to 65/500 (13.0%, 95% Wilson CI 10.3%‐16.2%), and best-of-128 achieved 0/500 (0%, 95% Wilson CI 0.0%‐0.8%)—matching the moderate *p*_valid_=0.12 regime. Across all 3 datasets, CTGAN+best-of-16 achieved 0/500 (0%) violations. For methods with 0/500 violations (eg, CTGAN+best-of-16 across all 3 datasets, WGAN-GP random sampling on Diabetes), the Wilson upper bound is 0.8% at n=500; for the stroke WGAN-GP variants with 500/500 violations, the Wilson lower bound is 99.2%.

Unfiltered CTGAN random sampling achieved substantially lower violation rates than WGAN-GP across all datasets (stroke: 226/500, 45.2%; 95% Wilson CI 40.9%‐49.6%; diabetes: 14/500, 2.8%; 95% Wilson CI 1.7%‐4.6%; cardiovascular: 11/500, 2.2%; 95% Wilson CI 1.2%‐3.9%), with strong distributional fidelity (KS mean 0.07‐0.12, correlation preservation >0.90). CTGAN+best-of-16 then reduced violations to 0/500 on all 3 datasets, improving TSTR AUC on stroke from 0.3787 (CTGAN random) to 0.4772. The purely unconstrained WGAN-GP baseline (λ=0) produced per-sample violation rates of 100.0% (500/500) on stroke, 0.6% (3/500; 95% Wilson CI 0.2%‐1.7%) on diabetes, and 0.0% (0/500; 95% Wilson CI 0.0%‐0.8%) on cardiovascular disease. This indicates that the moderate-valid support regime (*p*_valid_=0.12) is a characteristic of the penalized generator (λ=0.1) rather than the dataset itself. Adding the soft-constraint penalty (λ=0.1) to the WGAN-GP training loss degraded the model’s optimization stability, causing it to produce highly distorted distributions and a high violation rate (88.2%) on the cardiovascular dataset, whereas the unconstrained model (λ=0) avoided this training instability and directly learned the valid clinical ranges from the data. This comparison confirms that the soft-constraint penalty can degrade the baseline generator’s validity, making inference-time best-of-N selection necessary to recover validity in penalized models.

### Mechanistic Interpretation Through Joint Validity

**Figure 2.**
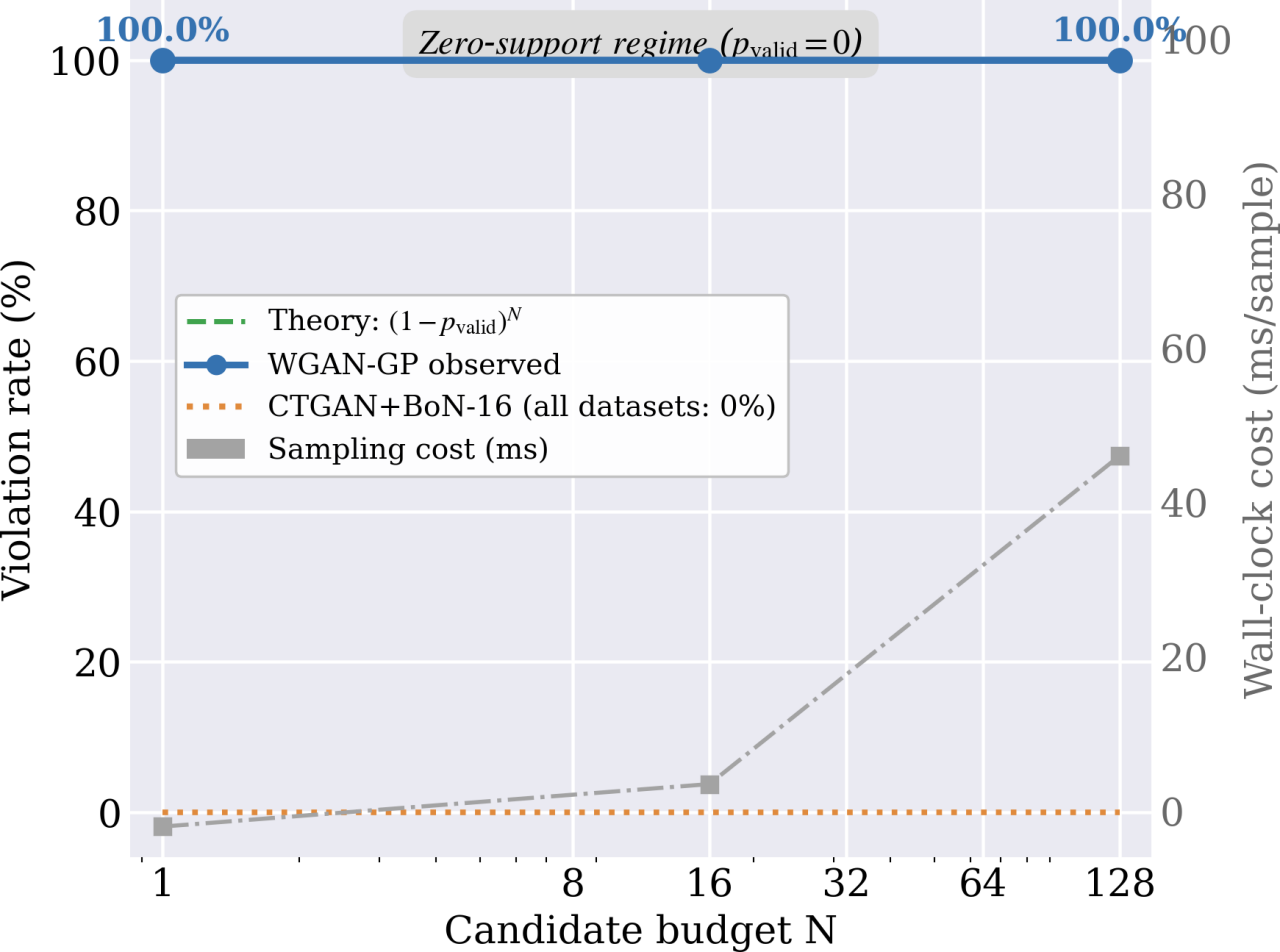
Stroke Wasserstein generative adversarial network with gradient penalty (WGAN-GP) scaling behavior. The violation curve remains flat at 100.0%, while cost rises with N, illustrating a zero-valid support regime in which selection cannot improve validity. BoN: best-of-N; CTGAN: conditional tabular generative adversarial network.

**Figure 3.**
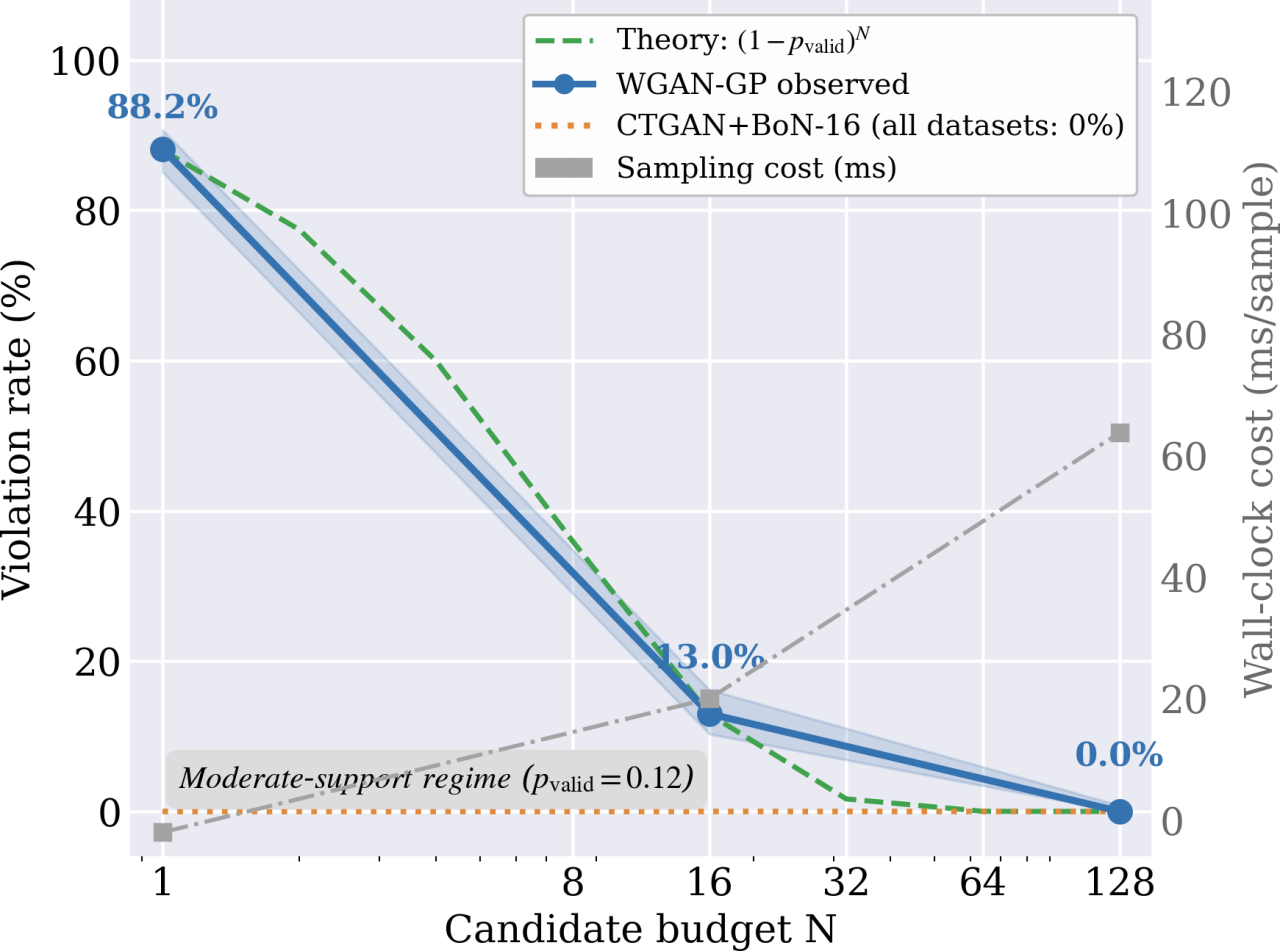
Cardiovascular Wasserstein generative adversarial network with gradient penalty (WGAN-GP) scaling behavior. Violation rate falls from 88.2% at n=1% to 13.0% at n=16% and 0.0% at n=128, while the sampling cost increases approximately linearly. BoN: best-of-N; CTGAN: conditional tabular generative adversarial network.

The stroke and cardiovascular results align closely with the joint-validity interpretation. For stroke WGAN-GP, the empirical valid support mass was *p*_valid_=0.0, yielding predicted success of 0.0 for N=8, N=16, and N=128, with observed success also being 0.0 in all cases (Figure 2). For cardiovascular WGAN-GP, *p*_valid_=0.12, and predicted success matched observed success almost exactly: 0.6404 vs 0.63 at N=8, 0.8707 vs 0.87 at N=16, and 1.0 vs 1.0 at N=128 (Figure 3). These results make the central point of the paper explicit: best-of-N cannot rescue a generator with zero-valid support mass, but when valid support mass is moderate, bounded-budget selection behaves as expected from the empirical value of *p*_valid_.

### Constraint Sensitivity and Rejection Comparisons

Wall-clock sampling costs scaled approximately linearly with N: a single random draw required 0.35 to 0.56 ms, best-of-16 cost 6.86 to 9.68 ms, and best-of-128 cost 46.59 to 67.89 ms across the 3 datasets.

Sensitivity analysis showed that moderate reweighting did not materially change total cardiovascular violation rates, whereas removing the gender term did. For cardiovascular data, best-of-16 with equal weights yielded 11.0% violations in the sensitivity pool; best-of-16 with severity weighting also yielded 11.0% violations; and best-of-16 without the gender term increased violations to 53.5%. For stroke, all weighting profiles remained at 100.0% invalid, indicating that the problem was insufficient valid support rather than poor weighting.

Strict rejection sampling clarified the difference between ranking selection and true acceptance filtering. In stroke, rejection failed completely, producing 0 valid records across 5000 draws, consistent with the zero-support interpretation. In cardiovascular data, strict rejection was feasible: it produced 100 valid records from 724 draws (acceptance rate 0.1381) with 6.02 ms per valid sample. This was faster per valid sample than best-of-16 (10.77 ms/valid) and much faster than best-of-128 (63.79 ms/valid), indicating that best-of-N should not be framed as uniformly superior to rejection sampling when *p*_valid_ is already moderate.

### Fidelity and Utility

**Figure 4.**
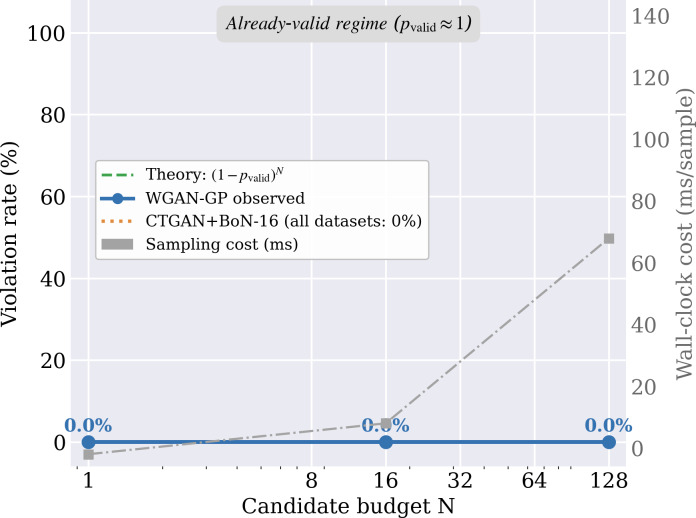
Diabetes N-scaling behavior. Violation rate remains at 0% for all Wasserstein generative adversarial network with gradient penalty (WGAN-GP) variants (already-valid regime), yet TSTR (train-on-synthetic-test-on-real) AUC (area under the receiver operating characteristic curve) rises steadily with N (0.5775 → 0.7149 → 0.7258), demonstrating that selection can shift the output distribution toward more useful regions even when constraint satisfaction is not the bottleneck. BoN: best-of-N; CTGAN: conditional tabular generative adversarial network.

**Figure 5.**
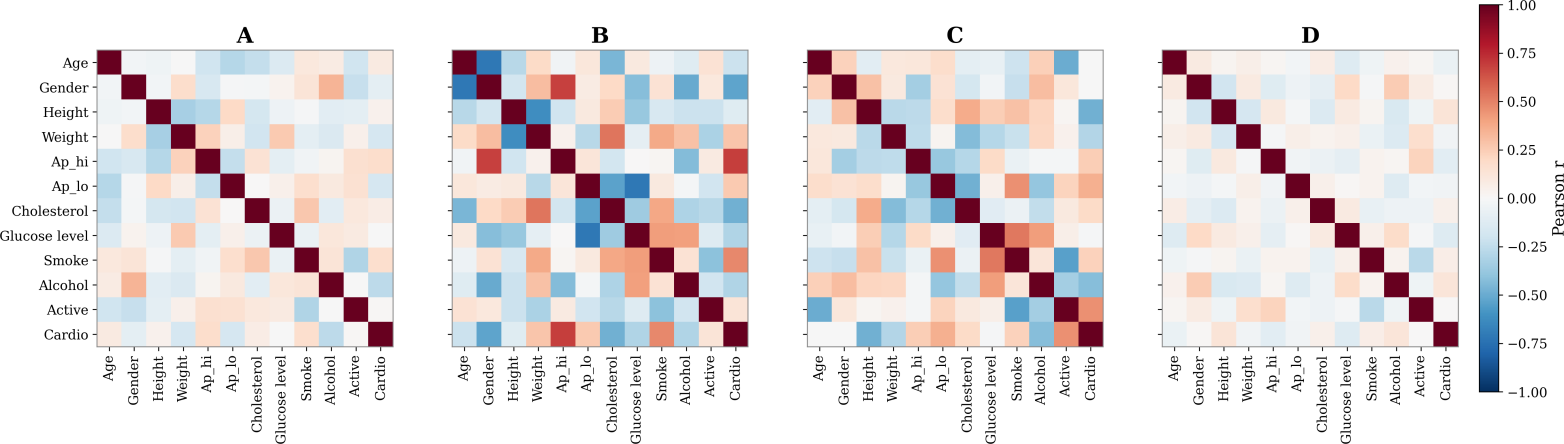
Cardiovascular correlation heatmaps. (A) Real training data. (B) The Wasserstein generative adversarial network with gradient penalty (WGAN-GP) random model exhibits severe structural distortion (λ=0.1, Frobenius norm=6.44), (C) WGAN-GP best-of-16 improves relational validity but remains far from the real correlation structure (Frobenius norm=4.59), and (D) conditional tabular generative adversarial network (CTGAN)+best-of-16 remains closest to the real matrix overall (Frobenius norm=1.36).

Table 3 summarizes fidelity and utility for the main comparator methods. Figure 4 shows the utility-shift pattern in the diabetes dataset, where best-of-N substantially improves TSTR AUC, even though the validity is already 100%. CTGAN was consistently stronger than WGAN-GP on distributional fidelity, with much lower KS statistics and lower Frobenius error in the correlation matrix. Figure 5 illustrates this for the cardiovascular dataset. On the diabetes dataset, WGAN-GP best-of-N improved utility despite no validity gain: TSTR AUC increased from 0.5775 for WGAN-GP random sampling to 0.7149 for best-of-16 and 0.7258 for best-of-128. On the cardiovascular dataset, WGAN-GP best-of-128 reached 0.0% violations but remained poor for utility (TSTR AUC 0.50), whereas CTGAN+best-of-16 achieved both 0.0% violations and strong utility (TSTR AUC 0.7368). On the stroke dataset, CTGAN+best-of-16 improved TSTR AUC from 0.3787 for CTGAN random sampling to 0.4772 while preserving near-zero KS discrepancy in the gender columns. Naive clipping failed on utility across all datasets: on diabetes, it produced 500/500 (100%) violations due to binary feature distortion, rendering TSTR evaluation meaningless; on stroke and cardiovascular data, it maintained marginal utility (TSTR AUC=0.5000) at the cost of substantially higher KS statistics. These results confirm that naive clipping is not a viable substitute for constraint-minimizing selection when both validity and distributional fidelity are required.

**Table 3.** Selected fidelity and utility results for the main comparator methods^a^.

Dataset and method	KS^b^, mean (SD)	Correlation preservation, mean (SD)	TSTR^c^ AUC^d^, mean (SD)	MIA^e^ AUC, mean (SD)
Stroke				
WGAN^f^ random	0.9161 (0.1137)	0.8118 (0.1810)	0.5000 (0.0000)	0.4997 (0.0180)
WGAN BoN^g^-16	0.9153 (0.1138)	0.8162 (0.1709)	0.5000 (0.0000)	0.4999 (0.0191)
Naive clip	0.5766 (0.4380)	0.8442 (0.1993)	0.5000 (0.0000)	0.4996 (0.0187)
CTGAN^h^ random	0.0938 (0.0604)	0.9023 (0.1041)	0.3787 (0.0463)	0.4555 (0.0174)
CTGAN BoN-16	0.0789 (0.0562)	0.8969 (0.0986)	0.4772 (0.0428)	0.4978 (0.0201)
Diabetes				
WGAN random	0.8989 (0.1397)	0.7325 (0.1780)	0.5775 (0.0103)	0.4902 (0.0184)
WGAN BoN-16	0.8971 (0.1406)	0.7203 (0.1802)	0.7149 (0.0112)	0.4894 (0.0176)
WGAN BoN-128	0.8979 (0.1400)	0.7370 (0.1765)	0.7258 (0.0118)	0.4899 (0.0173)
Naive clip	0.5040 (0.3301)	0.8522 (0.1899)	0.5000 (0.0000)	0.4896 (0.0199)
CTGAN random	0.1183 (0.1286)	0.9321 (0.0542)	0.7922 (0.0083)	0.4581 (0.0184)
CTGAN BoN-16	0.1198 (0.1206)	0.9366 (0.0544)	0.8075 (0.0095)	0.4657 (0.0192)
Cardiovascular				
WGAN random	0.8622 (0.1584)	0.5366 (0.2702)	0.5000 (0.0000)	0.5006 (0.0188)
WGAN BoN-16	0.8730 (0.1609)	0.6763 (0.2038)	0.5000 (0.0000)	0.5010 (0.0184)
WGAN BoN-128	0.8731 (0.1660)	0.7406 (0.1990)	0.5000 (0.0000)	0.5032 (0.0175)
Naive clip	0.8420 (0.1922)	0.5912 (0.2618)	0.5000 (0.0000)	0.5006 (0.0170)
CTGAN random	0.0711 (0.0359)	0.9185 (0.0721)	0.7260 (0.0073)	0.4980 (0.0168)
CTGAN BoN-16	0.0828 (0.0447)	0.9115 (0.0714)	0.7368 (0.0091)	0.5052 (0.0178)

a SD for KS mean and correlation preservation reflects variability across dataset features (or matrix entries); SD for TSTR AUC and MIA AUC reflects variability from bootstrap resampling (200 iterations) of a fixed trained model. These SD values characterize within-run variability rather than variability across independently retrained models with different random seeds.

b KS: Kolmogorov-Smirnov.

c TSTR: train-on-synthetic-test-on-real.

d AUC: area under the receiver operating characteristic curve.

e MIA: membership inference attack.

f WGAN: Wasserstein generative adversarial network.

g BoN: best-of-N.

h CTGAN: conditional tabular generative adversarial network.

### Privacy Results

**Figure 6.**
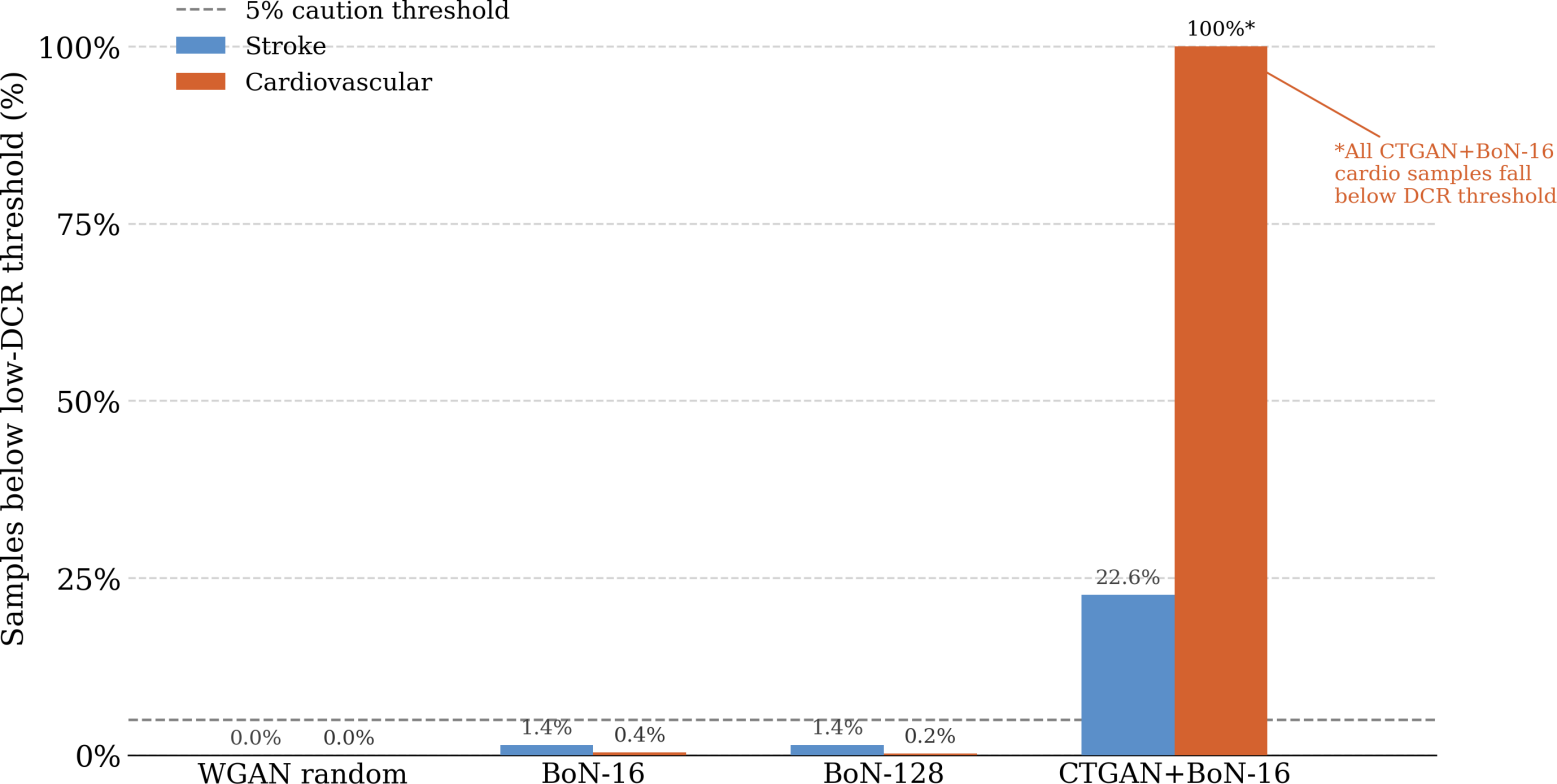
Distance to closest record (DCR) proximity concentration by method (stroke and cardiovascular datasets; diabetes omitted as concentration analysis was not applicable). Concentration measures the percentage of synthetic samples falling below the first-percentile DCR threshold of the random-sampling baseline. Wasserstein generative adversarial network with gradient penalty (WGAN-GP) variants remain near 0 across all N, while conditional tabular generative adversarial network (CTGAN)+best-of-16 reaches 22.6% for stroke and 100% for cardiovascular data, indicating that stronger generators combined with selection increase output proximity to training records even when membership inference attack (MIA) AUC (area under the receiver operating characteristic curve) remains near 0.50. BoN: best-of-N.

MIA AUC remained close to 0.50 for all methods and datasets, with no clear evidence of successful membership inference. Table 4 reports exact MIA AUC and DCR mean values; Figure 6 shows the DCR proximity concentration pattern. For WGAN-GP on stroke and cardiovascular data, best-of-N changed concentration only slightly (stroke: 1.4%; cardiovascular: 0.4%). CTGAN+best-of-16 showed markedly stronger DCR concentration (22.6% for stroke; 100.0% for cardiovascular), even though MIA remained near chance throughout. These results support a cautious interpretation: no membership inference leakage was detected by MIA, but proximity-based concentration warrants additional monitoring when applying selection to stronger generators.

**Table 4.** Privacy results for main methods^a^^,^^b^.

Dataset and method	MIA^c^ AUC^d^, mean (SD)	DCR^e^, mean (SD)
Stroke		
WGAN^f^ random	0.4997 (0.0180)	5.07 (1.24)
WGAN BoN^g^-16	0.4999 (0.0191)	4.97 (1.23)
CTGAN^h^ random	0.4555 (0.0174)	6.04 (4.48)
CTGAN BoN-16	0.4978 (0.0201)	5.01 (3.88)
Diabetes		
WGAN random	0.4902 (0.0184)	9.63 (1.17)
WGAN BoN-16	0.4894 (0.0176)	9.67 (1.17)
CTGAN random	0.4581 (0.0184)	2.71 (1.32)
CTGAN BoN-16	0.4657 (0.0192)	2.55 (1.00)
Cardiovascular		
WGAN random	0.5006 (0.0188)	25.93 (1.74)
WGAN BoN-16	0.5010 (0.0184)	25.09 (1.34)
WGAN BoN-128	0.5032 (0.0175)	24.99 (1.21)
CTGAN random	0.4980 (0.0168)	3.58 (2.88)
CTGAN BoN-16	0.5052 (0.0178)	3.18 (2.21)

a MIA AUC near 0.50 indicates no membership inference leakage. DCR mean indicates the average distance to the nearest real record (higher values indicate greater safety).

b SD for MIA AUC reflects variability from bootstrap resampling (200 iterations) of a fixed trained model. SD for DCR mean reflects variability across individual synthetic samples. These SD values characterize within-run variability rather than variability across independently retrained models with different random seeds.

c MIA: membership inference attack.

d AUC: area under the receiver operating characteristic curve.

e DCR: distance to closest record.

f WGAN: Wasserstein generative adversarial network.

g BoN: best-of-N.

h CTGAN: conditional tabular generative adversarial network.

## Discussion

### Principal Findings

Three findings emerge from these experiments. First, best-of-N functions as a constraint-minimizing selector whose effectiveness depends on the generator’s valid support mass. The stroke WGAN-GP model had *p*_valid_=0, and neither bounded-budget selection nor strict rejection across 5000 draws could recover valid samples. Second, when valid support mass is moderate (cardiovascular WGAN-GP, *p*_valid_=0.12), empirical best-of-N behavior closely follows the theoretical success probability 1 − (1 − *p*_valid_)^*N*^. Third, CTGAN+best-of-16 delivered the strongest practical validity-fidelity-utility trade-off across all 3 datasets. Regarding privacy, MIA AUC remained near random guessing (0.50) throughout all experiments, which is reassuring. However, DCR-threshold concentration analysis revealed that stronger generators, combined with selection, can shift outputs closer to the training data in distance-based terms—a cautionary signal that privacy should not be assessed from MIA alone when applying selection to high-capacity generators.

### Comparison With Prior Work

These findings refine the usual comparison between architectural constraint handling and post hoc filtering. Best-of-N is not a universal replacement for architectural modeling, but it can serve as a practical inference-time wrapper when the base generator already places nontrivial probability mass near the feasible region. Therefore, our results sharpen rather than weaken the contribution: inference-time selection is effective in a subset of regimes that can be characterized empirically through *p*_valid_, making the method more interpretable than a blanket “works across datasets” claim.

### Practical Implications

Generator quality matters more than candidate budget alone. WGAN-GP behaved differently across datasets: it failed completely on stroke data, was already valid on diabetes, and improved only gradually on cardiovascular data. CTGAN consistently produced low raw violation rates and responded well to best-of-16 selection. The primary recommendation from this study is therefore not simply to increase N but rather to pair bounded-budget selection with a generator that already models the feasible region reasonably well.

The rejection comparison clarifies an important boundary: when *p*_valid_ is 0, both rejection and best-of-N fail; when *p*_valid_ is moderate, strict rejection can be more efficient per valid sample than a large-budget best-of-N. Best-of-N’s practical value lies in its bounded computational overhead and compatibility with existing generators, not in guaranteed dominance over rejection sampling. On utility, mild best-of-N selection can shift the synthetic training distribution in a beneficial direction even when validity is already high (diabetes), but feasibility alone does not guarantee utility (cardiovascular: WGAN-GP best-of-128 achieved 0% violations, yet TSTR AUC remained 0.50).

### Limitations

First, this study is limited to 3 tabular clinical datasets and may not generalize directly to time series, imaging, or free-text medical data. Future work should assess generalizability across a wider range of EHR modalities. This may influence the generalizability of the 3-regime taxonomy, as datasets with richer temporal or unstructured constraint structures could yield qualitatively different *p*_valid_ distributions. Second, our diabetes validity definition follows the operational rule used in the code and is therefore a dataset-specific consistency heuristic rather than a universal clinical statement; the clinical significance of this rule should be validated by domain experts. As a result, the already-valid regime classification of diabetes may partly reflect the heuristic’s leniency rather than genuine generator validity, potentially overstating the method’s practical reach. Third, CTGAN concentration results indicate that stronger generators may require additional privacy diagnostics beyond MIA; practitioners should use DCR-threshold analysis alongside standard membership inference tests when deploying selection-augmented generators. The 100% DCR-threshold concentration for cardiovascular CTGAN +best-of-16 suggests that MIA AUC alone may substantially underestimate privacy risk, potentially giving practitioners a false sense of safety in high-stakes settings. Fourth, the paper reports one set of constraint weights as the default, and although sensitivity analyses reduced concern about arbitrariness, more comprehensive weighting strategies or lexicographic feasibility rules remain open for future work. Severity-weighted scoring could, in principle, produce different validity-utility trade-offs than those observed here, particularly in datasets where some constraint categories carry greater clinical consequence than others.

### Future Directions

Future work should explore adaptive policies that select among random sampling, rejection sampling, and best-of-N based on the estimated *p*_valid_; extend the same framework to diffusion- and language-model-based tabular generators [13,14]; and evaluate whether privacy-preserving training alters the interaction between valid support mass and inference-time selection.

### Conclusions

Best-of-N selection is useful when treated as a bounded-budget feasibility filter rather than a universal repair mechanism. The present experiments show that its success is governed by the generator’s valid support mass: when that mass is 0, both selection and rejection fail; when it is moderate, empirical gains align with theoretical expectations; and when the generator is already strong, selection can remove residual violations at modest additional cost. In our setting, CTGAN+best-of-16 offered the strongest overall practical result across clinical validity, fidelity, utility, and privacy.

## Abbreviations

AUC: area under the receiver operating characteristic curve
CorGAN: correlational generative adversarial network
CTGAN: conditional tabular generative adversarial network
DCR: distance to closest record
EHR: electronic health record
GAN: generative adversarial network
GDPR: General Data Protection Regulation
GReaT: Generation of Realistic Tabular data
HIPAA: Health Insurance Portability and Accountability Act
KS: Kolmogorov-Smirnov
LLM: large language model
MedGAN: medical generative adversarial network
MIA: membership inference attack
ROC: receiver operating characteristic
TSTR: train-on-synthetic-test-on-real
WGAN-GP: Wasserstein GAN with gradient penalty
